# ICU-Associated *Acinetobacter baumannii* Colonisation/Infection in a High HIV-Prevalence Resource-Poor Setting

**DOI:** 10.1371/journal.pone.0052452

**Published:** 2012-12-27

**Authors:** Ntobeko B. A. Ntusi, Motasim Badri, Hoosain Khalfey, Andrew Whitelaw, Stephen Oliver, Jenna Piercy, Richard Raine, Ivan Joubert, Keertan Dheda

**Affiliations:** 1 The Cardiac Clinic, Department of Medicine, Groote Schuur Hospital and University of Cape Town, Cape Town, South Africa; 2 Lung Infection and Immunity Unit, Division of Pulmonology & Clinical Immunology, Department of Medicine, Groote Schuur Hospital and University of Cape Town, Cape Town, South Africa; 3 Department of Critical Care, Groote Schuur Hospital and University of Cape Town, Cape Town, South Africa; 4 Department of Microbiology and National Health Laboratory Service, Groote Schuur Hospital and University of Cape Town, Cape Town, South Africa; University of Ottawa, Canada

## Abstract

**Background:**

There are hardly any data about the incidence, risk factors and outcomes of ICU-associated *A.baumannii* colonisation/infection in HIV-infected and uninfected persons from resource-poor settings like Africa.

**Methods:**

We reviewed the case records of patients with *A.baumannii* colonisation/infection admitted into the adult respiratory and surgical ICUs in Cape Town, South Africa, from January 1 to December 31 2008. In contrast to colonisation, infection was defined as isolation of *A.baumannii* from any biological site in conjunction with a compatible clinical picture warranting treatment with antibiotics effective against *A.baumannii*.

**Results:**

The incidence of *A.baumannii* colonisation/infection in 268 patients was 15 per 100 person-years, with an in-ICU mortality of 26.5 per 100 person-years. The average length of stay in ICU was 15 days (range 1–150). *A.baumannii* was most commonly isolated from the respiratory tract followed by the bloodstream. Independent predictors of mortality included older age (p = 0.02), low CD4 count if HIV-infected (p = 0.038), surgical intervention (p = 0.047), co-morbid Gram-negative sepsis (p = 0.01), high APACHE-II score (p = 0.001), multi-organ dysfunction syndrome (p = 0.012), and a positive blood culture for *A.baumannii* (p = 0.017). Of 21 *A.baumannii* colonised/infected HIV-positive persons those with clinical AIDS (CD4<200 cells/mm^3^) had significantly higher in-ICU mortality and were more likely to have a positive blood culture.

Conclusion In this resource-poor setting *A.baumannii* infection in critically ill patients is common and associated with high mortality. HIV co-infected patients with advanced immunosuppression are at higher risk of death.

## Introduction

Traditionally, *Acinetobacter baumannii* has been considered to be an opportunistic pathogen of low pathogenicity [1], but the emergence of serious community-acquired *A.baumannii* infection has demonstrated that this organism can be highly virulent with a propensity to cause invasive disease in non-critically ill patients. [2] Nosocomial transmission is responsible for the vast majority of *A.baumannii* infections, [3] and is related both to the ability of the organism to survive in the environment and the organism’s resistance to the major groups of antibiotics, resulting in a selective advantage in settings such as ICUs, where broad-spectrum antibiotic use is commonplace. The incidence of nosocomial infections due to Gram negative infections in South Africa has previously been reported to be between 24 and 36%. [4], [5] When *A.baumannii* is isolated from patient-derived biological samples distinction is often made between colonisation and active infection (associated features of sepsis and/or a positive blood culture). [6] Globally, there has been a general trend of increasing incidence of infection due to *A.baumannii*. [7] For instance, in France nosocomial infection due to *A.baumannii* was rare in the 1970s and had increased to a prevalence of 9% by 2005. [8] Similarly, in North America, the prevalence of nosocomial infection due to *A.baumannii* increased from 4% in 1986 to 7% in 2003. [9] Patients at risk are often critically ill with multiple co-morbidities, concurrent infections, and on prolonged courses of antibiotics, often making it difficult to distinguish between colonisation and infection. [10] Moreover, colonisation is a risk factor for subsequent infection [6].

Rational planning of health care policy and interventional measures are required to tackle ICU-associated *A.baumannii* infection in resource-poor settings. However, there are inadequate data on which to plan interventional strategies and base recommendations. Firstly, the frequency, risk factors and associated mortality of *A.baumannii* in African ICUs have not been determined. Thus, apart from a brief report describing the molecular epidemiology of *A.baumannii* [11] and the comparative efficacy of different antimicrobials [12] there is a paucity of data about *A.baumannii* from the African continent and other resource-poor settings. Secondly, the incidence, risk factors and prognosis of HIV-infected persons with *A.baumannii* requires clarification; such data have important implications for management of patients from high HIV prevalence settings. We hypothesised that, in contrast to developed countries, in resource-poor settings like South Africa *A.baumannii* colonisation/infection is less common but associated with altered outcomes in HIV-infected persons. The UNAIDS estimates that the prevalence of HIV infection in South Africa is 18%, [13] however the prevalence of HIV infected persons in adult ICUs in the country is generally much lower.

To address these questions we reviewed the case records of all patients with *A.baumannii* infection/colonisation admitted to the adult ICUs at Groote Schuur hospital (GSH) in Cape Town, South Africa. Cape Town has a population of ∼3.5 million people. GSH is a 900-bed, government-funded hospital providing tertiary care in all the major branches of medicine, and is the chief academic teaching hospital of the University of Cape Town. GSH employs 500 doctors, 1300 nurses and 250 allied health professionals. The public sector, within which GSH is situated, is over-subscribed and under-resourced, as it is constantly under pressure to deliver healthcare services to over 80% of the population, and faces the serious challenges of prevalent HIV/AIDS, poverty and shortage of medical personnel. The specific study aims were to (i) describe the incidence and site from which *A.baumannii* was isolated in an adult ICU population, (ii) determine the outcomes of patients with *A.baumannii* colonisation vs. infection, (iii) determine the risk factors associated with poor outcome in this subgroup of patients, and (iv) determine the epidemiology and prognosis of HIV-infected people with *A.baumannii* colonisation/infection.

## Methods

### Study Design

The study was designed as a retrospective cohort review of patients admitted into the adult ICUs at GSH.

### Study Population and Definitions

Patients from whom *A.baumannii* was isolated from any biological sample, from 1 January 2008 to 31 December 2008 following admission to one of the adult respiratory or surgical ICUs at GSH, were included in the study. The patients were identified from the National Health Laboratory System (NHLS) and the pharmacy computer records based on positive *A.baumannii* microbiology and/or treatment for *A.baumannii*.

*A.baumannii* infection was defined as any patient who had *A.baumannii* isolated from any site in conjunction with a compatible clinical picture warranting treatment with antibiotics effective against *A.baumannii*. The decision to treat was taken by the attending lead physician based on clinical signs and laboratory parameters indicating systemic inflammatory response/sepsis. Active infection cases were divided into definite (isolation from the bloodstream) or probable cases (isolation from another site, e.g. respiratory tract). Colonisation was defined as isolation of the organism with no antibiotic therapy as it was deemed by the attending physician not to be clinically significant.

This study was approved by the University of Cape Town Research Ethics Committee. However, written informed consent was not obtained from patients for the information to be stored into a hospital database and to be utilised for research as this is a retrospective study and the data was collected after patients had been discharged from the hospital. This conduct was approved by the Research Ethics Committee.

### Measurements and Data Collection

A comprehensive data collection database that incorporated patient demographic details, medical history and co-morbidity, as well as details of ICU stay and management was utilised. The specific patient-related risk factors that were explored included HIV status, co-morbid disease, presence of C-reactive protein (CRP), serum albumin and increased white cell count. The treatment-related factors that were explored included prior and current antibiotic use and choice, use of mechanical ventilation, and instrumentation during the ICU admission. Factors related to the route of entry to ICU and management before the index ICU admission were considered. The specific outcome measures evaluated included death in the ICU, length of ICU admission, duration of antibiotic treatment in ICU (colistin, meropenem or aminoglycoside), cure in those with infection, and colonisation.

### Statistical Analysis

Pearson’s chi-square or Fisher’s exact test were used to compare the relative frequency of characteristics between the two groups of patients. Cox’s binary logistical regression analysis was used for determining independent predictors of mortality in patients infected/colonised with *A.baumannii* in the adult ICUs.

## Results

### Incidence of *A.baumannii* Colonisation/infection in the Adult ICUs

268 patients with positive *A.baumannii* cultures were identified in the adult ICUs at GSH in 2008. In the same year, 1784 patients were admitted to the ICU, giving an incidence of *A.baumannii* colonisation/infection of 15 per 100 person-years. Of the 268 patients diagnosed with *A.baumannii* during the study period, 17 had either missing folders or incomplete information, and were consequently excluded from the analysis. A sensitivity analysis indicated that the demographic profile and HIV status of these 17 patients was similar to that of the 251 patients analysed and thus unlikely to have biased the analysis.

### ICU Mortality Associated with Microbiological Isolation of *A.baumannii*

71 patients with *A.baumannii* infection died during their admission into the ICU in this study. The ICU mortality associated with *A.baumannii* was 26.5 per 100 person-years. In the same year, the overall ICU mortality at GSH was 14.7 per 100 person-years. In-hospital mortality (before or after ICU) and 30-day mortality were not recorded.

### Demographic, Clinical and Laboratory Features and Risk Factors for *Acinetobacter* Colonisation/infection in Patients Admitted to Adult ICUs

Of 251 patients included in the analysis, 213 (84.9%) had *A.baumannii* infection and 38 (15.1%) had *A.baumannii* colonisation. Of the 213 patients with *Acinetobacter* infection, 91 (42.7%) had definite infection, and the remaining 122 (57.3%) had probable infection. Among patients who had *A.baumannii* isolated, risk factors for developing colonisation instead of infection included prior ICU admission (2.3 vs. 28.9%, p<0.001), invasive ventilation in that ICU admission (20 vs. 90.9%, p<0.001), admission to the trauma unit before current ICU admission (26.8 vs. 63.2%, p = 0.003) and endotracheal tube (ETT) insertion in the trauma unit (19.7 vs. 47.4%, p<0.001); (Table 1). Risk factors for *A.baumannii* infection included recent surgery in the current hospital admission (28.2 vs. 7.9%, p = 0.001), and ETT insertion either in surgical theatre (29.6 vs. 2.6%, p<0.001); (Table 1).

**Table 1:** Demographic characteristics and risk factors for *Acinetobacter baumannii* in patients with infection vs. colonisation admitted to adult ICUs at Groote Schuur Hospital.

Characteristic	*A.baumannii* infection(N = 213)	*A.baumannii* colonisation (N = 38)	P value
Age in years, mean ± SD	41.64±16.05	41.33±18.23	0.485
Male	146 (68.6)	24 (63.2)	0.327
HIV positive	20 (9.4)	1 (2.6)	0.201
For HIV infected: CD4 count, median (range)	221 (69–606)	130	0.009
Prior ICU admission, n (%)	5 (2.3)	11 (28.9)	<0.001
Ventilated in prior ICU admission, n (%)	1 (20)	10 (90.9)	<0.001
Ward before coming to ICU, n (%)			0.003
Medical A&E	26 (12.2)	3 (7.9)	0.003
Trauma Unit	57 (26.8)	24 (63.2)	0.003
Surgical wards	74 (34.7)	5 (13.2)	0.003
Medical wards	12 (5.6)	0 (0)	0.003
Gynaecology-Obstetrics wards	3 (1.4)	0 (0)	0.003
Secondary hospital ICU	36 (16.9)	5 (13.2)	0.003
Private hospital ICU	2 (0.9)	0 (0)	0.003
TBH ICU	3 (1.4)	1 (2.6)	0.003
Place of ETT insertion, n (%)			<0.001
Medical A&E	13 (6.1)	2 (5.3)	<0.001
Trauma Unit	42 (19.7)	18 (47.4)	<0.001
Surgical wards	3 (1.4)	9 (23.7)	<0.001
Medical wards	0 (0)	1 (2.6)	<0.001
Adult ICU	39 (18.3)	6 (15.8)	<0.001
Surgical theatre	63 (29.6)	1 (2.6)	<0.001
Secondary hospital ICU	34 (16)	0 (0)	<0.001
Private ICU	1 (0.5)	0 (0)	<0.001
No ETT in this admission	18 (8.4)	1 (2.6)	<0.001
Recent surgery in this admission, n (%)	60 (28.2)	3 (7.9)	0.001
Season during admission to ICU, n (%)			0.128
Summer	69 (32.4)	14 (36.8)	0.128
Autumn	49 (23)	7 (18.4)	0.128
Winter	52 (24.4)	6 (15.8)	0.128
Spring	43 (20.2)	11 (28.9)	0.128
Admitted to hospital in the last six months before this ICU admission, n (%)	45 (21.1)	8 (21.1)	0.505
MAE in ICU, n (%)	167 (78.4)	2 (5.3)	<0.001

HIV = human immunodeficiency virus, CD4 = cluster of differentiation 4, ICU = intensive care unit, TBH = Tygerberg Hospital, ETT = endotracheal tube.

### Site of *A.baumannii* Isolation in ICU Patients

The respiratory tract was the most common site of isolation of *A.baumannii*, accounting for 135 (53.8%) of all patients analysed, and 24 (63.2%) of the patients meeting the case definition for *A.baumannii* colonisation. Of those with respiratory tract *A.baumannii* isolation, 98 (72%) had ventilator-associated pneumonia. The majority of patients with *Acinetobacter* respiratory colonisation/infection were discharged from ICU alive. 91 patients had *A.baumannii* isolated from the blood, accounting for 65% of the patients who demised in ICU, as shown in Table 2. Colonisation/infection in the urine and wound sites was less common, and accounted for 8.4% off all *A.baumannii* isolates, and 34.2% of *A.baumannii* colonisation in the patients studied.

**Table 2:** Site of *Acinetobacter baumannii* colonisation/infection in patients admitted to the adult ICUs at Groote Schuur Hospital.

Site of infection	Patient dischargedalive (N = 180)	Patient died inICU (N = 71)	*Acinetobacter baumannii* infection(N = 213)	*Acinetobacter baumannii*colonization (N = 38)	*P value*
Blood only (%)	15 (8.3)	33 (46.5)	48 (22.5)	0 (0)	0.021
TA/BAL and blood (%)	40 (22.2)	18 (25.4)	57 (26.7)	1 (2.6)	0.108
TA/BAL only (%)	108 (60)	12 (16.9)	96 (45.1)	24 (63.2)	0.002
Urine and blood (%)	2 (1.1)	2 (2.8)	4 (1.9)	0 (0)	0.469
Urine only (%)	0 (0)	0 (0)	0 (0)	0 (0)	N/A
Wound/abscess and blood (%)	7 (3.9)	6 (8.5)	8 (3.8)	0 (0)	0.078
Wound/abscess only (%)	8 (4.4)	0 (0)	0 (0)	13 (34.2)	0.179

TA = tracheal aspirate, BAL = broncheo-alveolar lavage. P value refers to comparison of patients with active *A.baumannii* discharged alive from ICU vs. those who died in ICU. Wound/abscess sites include intra-abdominal swabs.

### Antibiotic Susceptibility Patterns of *A.baumannii* Isolated from Patients Admitted to Adult ICUs

The antibiotic susceptibility patterns are shown on Table 3. High rates of resistance to cotrimoxazole, ciprofloxacin, meropenem, ceftazidime, cefepime, gentamycin, amikacin and pipericillin-tazobactam were noted. Colistin remained the most effective drug for treatment of multidrug-resistant *A.baumannii*, as 97.3% of strains were susceptible to it. However, in this study, there were 2 patients with *Acinetobacter* infection that were resistant to all antibiotics, including colistin. Use of carbapenems (p = 0.043) and aminoglycosides (p = 0.003) before the ICU admission were associated with higher ICU mortality in those with *A.baumannii* infection. Isolation of multidrug-resistant *A.baumannii* (resistant to at least 3 classes of antibiotics) was associated with increased mortality (p = 0.004). There were significantly higher proportions of patients receiving colistin (59.2 vs. 24.4%, p = 0.008), a carbapenem (49.3 vs. 25%, p<0.001) and/or an aminoglycoside (28.2 vs. 15%, p = 0.020) to treat infection in the group of patients who died in the ICU. In the group of 38 patients who were merely colonised with *A.baumannii*, and did not receive antibiotics in the ICU, there were no deaths (p = 0.004) compared to those with infection; (Tables S1 and S2).

**Table 3:** Drug susceptibility patterns of *A.baumannii* isolated from patients admitted to adults ICUs at Groote Schuur Hospital.

Antibiotics	Resistant	Sensitive	Intermediatesensitivity
Cotrimoxazole (%)	22 (81.5)	5 (18.5)	0 (0)
Ciprofloxacin (%)	17 (70.8)	6 (25)	1 (4.2)
Ceftazidime (%)	13 (68.4)	4 (21)	2 (10.5)
Cefepime (%)	11 (61.1)	6 (33.3)	1 (5.6)
Tobramycin (%)	8 (47)	7 (41.2)	2 (11.8)
Gentamycin (%)	23 (85.2)	3 (11.1)	1 (3.7)
Amikacin (%)	17 (85)	3 (15)	0 (0)
Pipericillin-tazobactam (%)	107 (89.9)	9 (7.6)	3 (2.5)
Meropenem (%)	224 (89.2)	25 (10.0)	2 (0.8)
Colistin (%)	2 (2.7)	71 (97.3)	0 (0)
Colistin MIC, mean (range)	3.25 (2.5–6)	0.6 (0.064–4)	–

MIC = minimum inhibitory concentration.

### Length of Hospital Stay and Duration of Antibiotic Treatment for Patients with *A.baumannii* Colonisation/infection Admitted to Adult ICUs

The patients who achieved cure had a median pre-ICU hospital stay of 2 days, and received antibiotics, usually given on an empiric basis, before admission to ICU, for a median of 2 days. The patients who died in ICU had a longer median pre-ICU hospital admission of 10 days (p<0.001) compared to those who achieved cure, and were also treated with antibiotics for longer before going to ICU [median 10 days (p<0.001)]. The median length of ICU stay was 9 days and 10 days for those who achieved cure and for those who died in ICU, respectively (p = 0.471). The median duration of ICU antibiotics was 7 days for both those who achieved cure and those who demised in ICU (p = 0.802); (Table S3).

### Risk Factors for Mortality in Patients with *A.baumannii* Infection Admitted to Adult ICUs

Patients who died from *A.baumannii* infection, when compared to those who survived, were more likely to be older (46 years vs. 38 years; p = 0.001), female (45.1% vs. 27.2%; p = 0.005), and HIV-infected (16.9% vs. 5.0%; p = 0.008); (Table S3). Dialysis, use of inotropes (for septic shock), development of multi-organ dysfunction syndrome [MODS] and cardiorespiratory arrest were more commonly associated with increased mortality in the ICU (p<0.001).

### Independent Predictors of Mortality in those with *A.baumannii* Colonisation/infection in the ICU

The risk factors for mortality from *A.baumannii* in those with HIV infection and in those with low CD4 counts are presented in Tables S4 and S5. Multivariate logistic regression analysis revealed that the independent predictors of mortality from *A.baumannii* infection were: older age (p = 0.023), low CD4 count if HIV positive (p = 0.038), surgery in the index admission (p = 0.047), Gram negative other than *A.baumannii* (p = 0.014), a higher APACHE II score (p = 0.001); MODS after *A.baumannii* infection (p = 0.012), and a positive blood culture result for *A.baumannii* (p = 0.017); (Table 4).

**Table 4:** Multivariate logistical regression analysis of predictors of mortality in those with *A.baumannii* colonisation/infection.

*Characteristic*	*Multi-variate logistic regression analysis*	
	Odds ratio (95% CI)	P-value
Age	1.073 (1.010–1.140)	0.023
If HIV positive, CD4 count	1.698 (1.042–3.275)	0.038
Recent surgery in this admission	5.942 (1.026–34.413)	0.047
Co-morbid Gram negative sepsis	11.494 (1.639–80.580)	0.014
APACHE II score	123.941(5.483–1373.197)	0.001
MODS after *A.baumannii* infection	83.003 (6.873–739.037)	0.012
Blood culture positive for *A.baumannii*	13.158 (1.595–108.522)	0.017

HIV = human immunodeficiency virus, CD4 = cluster of differentiation 4, APACHE II = Acute Physiology and Chronic Health Evaluation II, MODS = multi-organ dysfunction syndrome.

### HIV and *A.baumannii* Colonisation/infection

Of the 251 patients included in the analysis, 183 patients were HIV-uninfected, 47 patients’ HIV status was unknown and 21 patients were HIV-infected. When comparing the HIV infected to the uninfected patients, the predictors of *Acinetobacter* colonisation/infection included younger age (p = 0.042), female gender (p = 0.028), CD4 count below 200 cells/ml (p = 0.037), an APACHE II score greater than 30 (p = 0.010), and MODS (p = 0.032); (Table 5). As shown in Table 6, on multivariate logistic regression analysis, the specific predictors of mortality in HIV patients with CD4 counts below 200 cells/ml (compared to those above this threshold) were the occurrence of MAEs in ICU (p = 0.023), development of MODS (p = 0.041) and an APACHE II score greater than 30 (p = 0.001). The conclusions of this analysis did not change significantly when the analysis was confined to those with infection only vs. colonisation/infection.

**Table 5:** Multivariate logistical regression analysis of features predictive for Acinetobacter infection in HIV-infected persons when compared to HIV-uninfected patients.

*Characteristic*	*Multi-variate logistic regression analysis*	
	Odds ratio (95% CI)	P-value
Younger age	1.206 (1.013–1.638)	0.042
Female gender	1.607 (1.230–2.998)	0.028
CD4 count <200 cells/ml	3.878 (1.475–12.943)	0.037
Elevated APACHE II score (>30)	7.232 (1.567–18.479)	0.010
MODS after *A.baumannii* infection	9.573 (3.265–113.672)	0.032

**Table 6:** Multivariate logistical regression analysis of predictors of mortality in HIV-infected persons with Acinetobacter infection and a CD4 count <200 vs. >200 cells/ml.

*Characteristic*	*Multi-variate logistic regression analysis*	
	Odds ratio (95% CI)	P-value
MODS after *A.baumannii* infection	13.001 (2.763–57.984)	0.041
APACHE II score (>30)	21.073 (6.839–137.852)	0.001

## Discussion

To our knowledge this is the first detailed report describing *A.baumannii* epidemiology and outcomes from a resource-poor, high HIV prevalence setting. The key findings of our study were that ICU-associated *A.baumannii* infection in this setting: (i) occurs frequently; (ii) has a high in-ICU mortality; (iii) is frequently multidrug-resistant but remains largely susceptible to colistin; (iv) has a predilection for the respiratory tract and bloodstream, and often occurs in conjunction with ventilator-associated pneumonia, and (v) death in patients with *A.baumannii* infection is associated with several parameters including older age, low CD4 count if HIV positive, surgery during admission, co-morbid Gram negative sepsis, a high APACHE II score, and a positive blood culture for *A.baumannii*. Importantly, this is the first study to show that in those with ICU-related *A.baumannii* infection advanced HIV infection was associated with higher mortality. Our results have implications for resource allocation and management of patients with *A.baumannii* colonisation/infection in resource-poor settings, and highlight the need for aggressive treatment of patients with a positive blood culture, who are older, have a low CD4 count in the context of HIV-infection, high APACHE II scores at admission, or those with evidence of MODS.

The incidence and prevalence of *A.baumannii* infection is on the rise in Western nations. [14]–[17] By contrast, although thought to be an uncommon pathogen in an African setting, we show here that *A.baumannii* colonisation/infection is common in a South African ICU with an incidence of 15 per 100 person-years. Mortality was also high at 26.5 per 100 person-years. The mortality is similar to that reported by Trottier et al of 26.2% in critically ill American patients. [18] Other reports have demonstrated a mortality rate of 8–43% due to *A.baumannii* in Western countries [19]–[21] and of 33–45% in developing countries outside Africa, however, several of these studies did not control for confounding risk factors including age, disease severity, co-morbidity etc. [22], [23] We found a similar incidence and mortality rate to resource rich countries, where control of *A.baumannii* remains a significant challenge. Thus, solutions to the challenge of managing *A.baumannii* are likely to transcend resource and economic boundaries, and should include strict attention to infection control, stringent antibiotic stewardship, molecular fingerprinting to uncover transmission links, and development of novel strategies including therapeutic immunisation against the bacterium. [24] However, as Perez and colleagues note, “the success of all of these approaches depends on the commitment of clinical practitioners, scientists, hospital and public health administrators and on the support of an informed and concerned public.” [24].

There are hardly any data about *A.baumannii* colonisation/infection in HIV-infected persons and none from high HIV prevalent settings like Africa. In a series of 10 patients, described over a 9-year period in Italy, advanced immunosuppression was associated with septicaemia. [22] While immunosuppression in general has been shown to be a significant risk factor for mortality from *A.baumannii* infection, [25] two studies with small numbers of HIV-infected patients have concluded that HIV infection does not predict poor outcomes from *A.baumannii* infection, as AIDS in these studies showed good outcome with adequate treatment. [18], [25] By contrast, we observed that HIV with clinical AIDS was associated with higher overall mortality and within the HIV-infected subgroup those with AIDS are more likely to have a positive blood culture. Moreover, compared to HIV-infected patients with preserved immunity, AIDS patients who died in the ICU were also younger, more likely to be female, sicker (higher APACHE II scores), and had more frequent MAEs and MODS. In contradistinction with our findings, multivariate logistical regression analysis from the large, multi-centre and multi-national EPIC II study showed that HIV infection was independently associated with a higher risk of *A.baumannii* infection, but not with increased mortality. [12] Our findings thus have implications for the management of HIV-infected patients with *A.baumannii* infection (signs of sepsis or a positive blood culture) and now need confirmation in larger prospective studies. First, it is important to treat *Acinetobacter* bacteraemia early with antibiotics. Second, HIV infected patients and in particular those with low immunity, are at increased risk of *A.baumannii* infection and mortality, and hence need to be treated aggressively in order to avert poor outcomes. Third, more research and resources are needed in order to develop and implement preventative strategies for *A.baumannii* infection, especially in those who are immunocompromised.

Risk factors for *A.baumannii* colonisation in this high HIV-prevalence setting included prior ICU admission, invasive ventilation in ICU, admission to the trauma unit before ICU admission and ETT insertion in the trauma unit. These data suggest that the trauma unit and ICUs themselves are likely reservoirs facilitating transmission, and targeted surveillance and eradication may be required in these areas. Nevertheless, although admission via the trauma unit was an important risk factor for colonisation, this group had minimal mortality presumably because they were young and with little co-morbidity. It is likely that *A.baumannii* in this study was largely nosocomially acquired as it was cultured in the second week of hospital admission in almost all patients and had a high intrinsic resistance to most broad-spectrum antibiotics.

This study had several limitations, including the inherent drawbacks of a retrospective design, variable dosing and duration of antibiotic therapy, simultaneous administration of other antibiotics, and lack of monitoring of resistance patterns before and after therapy. Physician bias in initiating antibiotic treatment (and hence subjective definition of colonisation) is a drawback that we are unable to address because of study design, however, our study conclusions did not change when the analysis was performed using those with infection rather than colonisation/infection. Moreover, it is likely that the physician-based classification has reasonable credibility given that there were no deaths in the colonisation group. A second major limitation of our study is the lack of a matched control population who did not have Acinetobacter colonisation/infection and thus we are unable to determine to what extent death was due to Acinetobacter rather than other co-morbidities. Nevertheless, mortality (26%) was higher than that generally found in the ICU (∼15%) and we were able to address our primary aim – to provide a preliminary description of the epidemiology, clinical profile and risk factors associated with mortality in HIV-infected compared to HIV uninfected persons in this resource-poor high HIV prevalence setting. Seventeen (6.3%) of the patients admitted to ICU with *A.baumannii* colonisation/infection during the study period either had missing files or incomplete information; however, a sensitivity analysis showed that this group was similar to those patients included in the study. Whether HIV per se is a risk factor for *A.baumannii* colonisation/infection cannot be determined within the current study design and will be addressed in a prospective study, which is now required in different African centres to confirm our findings.

In conclusion, our data show that *A.baumannii* colonisation/infection is frequent in critically ill patients admitted to ICUs in Cape Town, South Africa, and is associated with significant risk of death. Amongst those with *A.baumannii* colonisation/infection HIV-infection with clinical AIDS is associated with high mortality and *A.baumannii*-specific septicaemia. Collectively these data, including the description of risk factors for colonisation and mortality in HIV-infected and uninfected patients in the ICU, will likely facilitate the rational allocation of resources and implementation of targeted interventions to prevent and treat *A.baumannii* in high HIV-prevalence settings like South Africa.

## Supporting Information

Table S1 (DOC)

Table S2 (DOC)

Table S3 (DOC)

Table S4 (DOC)

Table S5 (DOC)
