# Supplementary material for: ICU-Associated Acinetobacter baumannii Colonisation/Infection in a High HIV-Prevalence Resource-Poor Setting
Source: PLoS One. 2012 Dec 27;7(12):e52452. doi: 10.1371/journal.pone.0052452 (PMC3531465; doi:10.1371/journal.pone.0052452)
Supplement: Table S1 — (DOC) [file pone.0052452.s001.doc]

Table S1: Clinical characteristics as predictors of mortality in patients with Acinetobacter baumannii colonisation/infection admitted to adult ICUs

| Characteristic | Patient discharged alive (N=180) | Patient died in ICU (N=71) | P value |
| --- | --- | --- | --- |
| Mean heart rate*in bpm ± SD | 109.76 ± 25.07 | 112.46 ± 24.71 | 0.440 |
| Mean systolic blood pressure* in mmHg ± SD | 117.30 ± 26.17 | 118.51 ± 29.38 | 0.751 |
| Mean diastolic blood pressure*in mmHg ± SD | 68.99 ± 18.14 | 66.74 ± 17.36 | 0.372 |
| Median temperature*in degrees Celsius (IQR) | 37 (36.42-37.92) | 38 (36.72-39) | <0.001 |
| Median respiratory rate* in bpm (IQR) | 18 (14-24) | 20 (16-25.25) | 0.133 |
| Increased volume of secretions*, n (%) | 16 (8.9) | 15 (21.1) | 0.041 |
| Mean GCS* ± SD | 13.65 ± 2.66 | 12.56 ± 1.10 | 0.102 |
| Median creatinine* in µmol/l (IQR) | 77.50 (57.75-131) | 157.50 (93.75- 343.5) | <0.001 |
| Mean CRP*in mg/dl ± SD | 123.35 ± 119.90 | 165.09 ± 133.81 | 0.483 |
| Median white cell count* in x10^6^ cells/ml (IQR) | 13.20 (10.62-17.03) | 15.08 (8.10-23.73) | 0.053 |
| Mean haemoglobin*in g/dl ± SD | 10.73 ± 2.29 | 10.00 ± 2.52 | 0.037 |
| Albumin*, g/l ± SD | 28.77 ± 8.26 | 25.23 ± 6.95 | 0.043 |
| Median total bilirubin* in mmol/l (IQR) | 11.0 (7.0-16.5) | 16.5 (8.0-93.0) | 0.041 |
| Mean pO2*in mmHg ± SD | 16.07 ± 7.13 | 16.43 ± 7.53 | 0.728 |
| Mean pCO2*in mmHg ± SD | 5.51 ± 2.07 | 4.98 ± 1.74 | 0.063 |
| Median O2 saturation*in % (IQR) | 98 (96-100) | 98 (96.25-99) | 0.301 |
| Indwelling catheter, n (%) | 178 (98.9) | 69 (97.2) | 0.318 |
| Positive blood culture result for A.baumannii, n (%) | 50 (27.8) | 41 (57.7) | <0.001 |
| Discoloured or bloody tracheal aspirate, n (%) | 129 (71.6) | 49 (69.0) | 0.538 |
| Positive tracheal aspirate culture result for A.baumannii, n (%) | 146 (81.1) | 50 (70.4) | 0.056 |
| Positive urine culture result for A.baumannii, n (%) | 2 (1.1) | 2 (2.8) | 0.322 |
| Positive A.baumannii culture obtained from another site, n (%) | 13 (7.2) | 6 (8.5) | 0.347 |
| APACHE II score, n ± SD | 17.17 ± 7.88 | 35.78 ± 10.14 | <0.001 |
| Co-morbidity, n (%) Cardiac pathology Respiratory pathology Trauma Liver failure GIT pathology Pancreatitis Chronic kidney disease Acute kidney injury requiring dialysis Gram negative sepsis Autoimmune conditions Dermatologic emergency Vascular pathology Neurological pathology Malignancy Tuberculosis | 29 (16.1) 27 (15.0) 92 (51.1) 0 (0.0) 5 (2.8) 4 (5.6) 14 (7.8) 35 (19.4) 31 (17.2) 3 (1.7) 1 (0.6) 3 (1.7) 3 (1.7) 9 (5.0) 7 (3.9) | 9 (12.7) 15 (21.1) 10 (14.1) 2 (2.8) 2 (2.8) 7 (3.9) 10 (14.1) 54 (76.1) 32 (45.1) 2 (2.8) 2 (2.8) 1 (1.4) 4 (5.6) 15 (21.1) 9 (12.7) | 0.318 0.162 <0.001 0.079 0.636 0.379 0.101 <0.001 <0.001  0.437 0.194  0.682 0.102  <0.001 0.014 |
| MAE in ICU, n (%) | 100 (55.6) | 69 (97.2) | <0.001 |
| MODS, n (%) | 72 (40.0) | 69 (97.2) | <0.001 |
| Concurrent infection, n (%) | 72 (40.0) | 56 (78.9) | <0.001 |
| Instrumentation in ICU, n (%) No instrumentation Pulmonary/bronchoscopic Cardiac (Cath, TOE) GIT (endoscopy) Urological ENT Orthopaedic Neurological | 145 (80.6) 16 (8.9) 3 (1.7) 4 (2.2) 3 (1.7) 5 (2.8) 4 (2.2) 0 (0) | 56 (78.9) 8 (11.3) 2 (2.8) 2 (2.8) 0 (0) 0 (0)  0 (0) 3 (4.2) | 0.464 |

GCS = , Glasgow Coma Score, CRP = C reactive protein, pO2 = Oxygen partial pressure, pCO2 = Carrbon dioxide partial pressure, APACHE II = Acute Physiology and Chronic Health Evaluation II, MAE = Major adverse events, MODS = Multi organ dysfunction syndrome, GIT = Gastrointestinal tract, ENT = Ear, nose and throat.

*Indicates initial measurements done on admission to the ICU.
