# Supplementary material for: ICU-Associated Acinetobacter baumannii Colonisation/Infection in a High HIV-Prevalence Resource-Poor Setting
Source: PLoS One. 2012 Dec 27;7(12):e52452. doi: 10.1371/journal.pone.0052452 (PMC3531465; doi:10.1371/journal.pone.0052452)
Supplement: Table S2 — (DOC) [file pone.0052452.s002.doc]

Table S2: Management and complications as determinants of mortality in patients with Acinetobacter baumannii colonisation/infection admitted to adult ICUs

| Outcome measure after A.baumannii infection | Patient discharged alive (N=180) | Patient died in ICU  (N=71) | P value |
| --- | --- | --- | --- |
| Death in ICU | 180 (71.7) | 71 (28.3) | N/A |
| Complications related A.baumannii, n (%) | 56 (31.1) | 66 (93) | <0.001 |
| Acute kidney injury requiring dialysis, n (%) | 35 (19.4) | 54 (76.1) | <0.001 |
| Acute respiratory distress syndrome, n (%) | 7 (3.9) | 28 (39.4) | <0.001 |
| Required inotropic support, n (%) | 54 (30) | 64 (90.1) | <0.001 |
| DIC/Coagulopathy, n (%) | 4 (2.2) | 22 (31) | <0.001 |
| Median duration of ICU antibiotics in days (IQR) | 7 (4-12) | 7 (4-14) | 0.802 |
| Merely colonised, n (%) | 38 (21.1) | 0 (0) | <0.001 |
| No antibiotics used in this hospital stay, n (%) | 16 (8.9) | 0 (0) | 0.004 |
| Treated with colistin, n (%) | 44 (24.4) | 29 (59.2) | 0.008 |
| Treated with carbapenem, n (%) | 45 (25) | 35 (49.3) | <0.001 |
| Treated with aminoglycoside, n (%) | 27 (15) | 20 (28.2) | 0.020 |
| Treated with fluoroquinolone, n (%) | 17 (9.4) | 7 (9.9) | 0.543 |
| Treated with vancomycin, n (%) | 14 (7.8) | 6 (8.5) | 0.519 |
| Treated with cotrimoxazole, n (%) | 21 (11.7) | 6 (8.5) | 0.311 |
| Treated with pipericillin-tazobactam, n (%) | 80 (44.4) | 39 (54.9) | 0.087 |
| Treated with tobramycin, n (%) | 13 (7.2) | 4 (5.6) | 0.446 |
| Treated with augmentin (co-amoxiclav), n (%) | 73 (35.2) | 24 (40.6) | 0.263 |
| Treated with penicillin, n (%) | 18 (10) | 10 (14.1) | 0.237 |
| Treated with cephalosporin, n (%) | 10 (5.6) | 2 (2.8) | 0.290 |
| Treated with metronidazole, n (%) | 7 (3.9) | 4 (5.6) | 0.379 |
| Treated with clindamycin, n (%) | 4 (2.2) | 4 (5.6) | 0.161 |
| Treated with clarithromycin, n (%) | 11 (6.1) | 6 (8.5) | 0.339 |
| Median length of ICU stay in days (IQR) | 9 (5-20) | 10 (4-18.5) | 0.471 |
