# Supplementary material for: ICU-Associated Acinetobacter baumannii Colonisation/Infection in a High HIV-Prevalence Resource-Poor Setting
Source: PLoS One. 2012 Dec 27;7(12):e52452. doi: 10.1371/journal.pone.0052452 (PMC3531465; doi:10.1371/journal.pone.0052452)
Supplement: Table S3 — (DOC) [file pone.0052452.s003.doc]

Table S3: Risk factors for mortality in patients with Acinetobacter baumannii colonisation/ infection admitted to adult ICUs

| Characteristic | Patients discharged alive (N=180) | Patient died in ICU (N=71) | P value |
| --- | --- | --- | --- |
| Age in years, median (IQR) | 38 (24-51) | 46 (36-61) | <0.001 |
| Male | 131 (72.8) | 39 (54.9) | 0.005 |
| HIV positive | 9 (5) | 12 (16.9) | 0.008 |
| For HIV infected: CD4 count, median (range) | 155 (69-524) | 74 (8-606) | 0.002 |
| For HIV infected: use of HAART, n (%) | 2 (22.2) | 4 (33.3) | 0.477 |
| Prior ICU admission, n (%) | 13 (7.2) | 3 (4.2) | 0.287 |
| Ward before coming to ICU, n (%) Medical A&E Trauma Unit Surgical wards Medical wards Gynaecology-Obstetrics wards Secondary hospital ICU Private hospital ICU TBH ICU | 18 (10) 73 (40.6) 53 (29.4) 3 (1.7) 2 (1.1) 26 (14.4) 1 (0.6) 4 (2.2) | 11 (15.5) 8 (11.3) 26 (36.6)  9 (12.7) 1 (1.4) 15 (21.1) 1 (1.4) 0 (0) | 0.037 |
| Intubated before coming to ICU, n (%) | 147 (81.7) | 41 (57.7) | <0.001 |
| Place of ETT insertion, n (%) Medical A&E Trauma Unit Surgical wards Medical wards Adult ICU Surgical theatre Secondary hospital ICU Private ICU No ETT in this admission | 9 (5) 54 (30.2) 6 (3.4) 0 (0)  21 (11.7) 48 (26.8) 28 (15.6)  0 (0) 13 (7.3) | 6 (8.5) 6 (8.5) 6 (8.5) 1 (1.4) 24 (33.8) 16 (22.5)  6 (8.5) 1 (1.4) 6 (8.5) | 0.018 |
| Recent surgery in this admission, n (%) | 142 (78.9) | 46 (64.8) | 0.017 |
| Duration of antibiotics in ward before admission to ICU in days, median (IQR) | 2 (1-6.25) | 10 (1-13.5) | <0.001 |
| Length of hospital stay in this admission before going to the ICU, median (IQR) | 2 (1-5.5) | 10 (2-14.75) | <0.001 |
| Admitted to hospital in the last six months before this ICU admission, n (%) | 37 (20.6) | 16 (22.5) | 0.425 |
| Timing of A.baumannii infection, days after hospital admission ± SD | 8.3 ± 2.7 | 9.1 ± 2.9 | 0.734 |

HIV = human immunodeficiency virus, CD4 = cluster of differentiation 4, ICU = intensive care unit, TBH = Tygerberg Hospital, ETT = endotracheal tube.
