# Supplementary material for: ICU-Associated Acinetobacter baumannii Colonisation/Infection in a High HIV-Prevalence Resource-Poor Setting
Source: PLoS One. 2012 Dec 27;7(12):e52452. doi: 10.1371/journal.pone.0052452 (PMC3531465; doi:10.1371/journal.pone.0052452)
Supplement: Table S4 — (DOC) [file pone.0052452.s004.doc]

Table S4: Colonisation vs. Acinetobacter infection in HIV-infected and HIV-uninfected patients admitted to adult ICUs

| Characteristic | **HIV-infected patients** | | **HIV-uninfected patients** | | **P value** |
| --- | --- | --- | --- | --- | --- |
|  | Infection  (N=20) | Colonised  (N=1) | Infection  (N=193) | Colonised  (N=37) |  |
| Age in years, median (IQR) | 25 (22-35) | 27 | 42 (36-59) | 44 (35-64) | 0.018 |
| Male | 7 (35) | 0 (0) | 141 (73.1) | 29 (78.4) | <0.001 |
| For HIV infected: CD4 count, median (range) | 221 (69-606) | 130 | N/A | N/A | N/A |
| Prior ICU admission, n (%) | 2 (10) | 1 (100) | 3 (1.6) | 9 (24.3) | 0.087 |
| Ward before coming to ICU, n (%) Medical A&E Trauma Unit Surgical wards Medical wards Gynaecology-Obstetrics wards Secondary hospital ICU Private hospital ICU TBH ICU | 4 (20) 0 (0) 5 (25) 3 (15) 1 (5) 7 (35) 0 (0) 0 (0) | 1 (0) 0 (0)  0 (0) 0 (0)  0 (0) 0 (0)  0 (0) 0 (0) | 22 (11.4) 57 (29.5) 69 (35.8) 9 (4.7) 2 (1.0) 29 (15.0) 2 (1.0) 3 (1.6) | 2 (5.4) 24 (64.9) 5 (13.5) 0 (0) 0 (0) 5 (13.5) 0 (0) 1 (2.7) | 0.042 |
| Intubated before coming to ICU, n (%) | 15 (75) | 1 (100) | 138 (71.5) | 34 (91.9) | 0.491 |
| Length of hospital stay in this admission before going to the ICU, median (IQR) | 7 (2-10) | 4 (0) | 7 (3-11) | 7 (3-10) | 0.763 |
| Recent surgery in this admission, n (%) | 2 (10) | 0 (0) | 58 (30.1) | 3 (8.1) | 0.256 |
| Admitted to hospital in the last six months before this ICU admission, n (%) | 3 (15) | 1 (100) | 42 (21.8) | 7 (18.9) | 0.378 |
| Timing of A.baumannii infection, median days after hospital admission (IQR) | 8 (5-12) | 9 (0) | 8 (5-14) | 9 (6-13) | 0.512 |
| Length of ICU stay in this admission in days, median (IQR) | 9 (6-20) | 14 (0) | 9 (5-21) | 10 (5-18) | 0.490 |
| Major adverse events in ICU, n (%) | 15 (75) | 0 (0) | 152 (78.8) | 2 (5.4) | 0.316 |
| Multi-organ dysfunction syndrome, n(%) | 16 (80) | 0 (0) | 104 (53.9) | 8 (21.6) | 0.012 |
| APACHE II score, n ± SD | 38.32 ± 14.7 | 21 ± 0 | 34.17 ± 12.3 | 16.10 ± 6.9 | 0.048 |
| Positive blood culture result for A.baumannii, n (%) | 11 (55) | 0 (0) | 94 (48.7) | 0 (0) | 0.423 |
| Positive tracheal aspirate culture result for A. baumannii, n (%) | 13 (65) | 1 (100) | 140 (72.5) | 24 (64.9) | 0.307 |
| Positive urine culture result for A.baumannii, n (%) | 1 (5) | 0 (0) | 3 (1.6) | 0 (0) | 0.785 |
| Positive A.baumannii culture from another site, n (%) | 1 (5) | 0 (0) | 7 (3.6) | 13 (35.1) | 0.105 |
| Deaths in ICU in this admission, n (%) | 12 (60) | 0 (0) | 59 (30.6) | 0 (0) | 0.014 |
