# Supplementary material for: ICU-Associated Acinetobacter baumannii Colonisation/Infection in a High HIV-Prevalence Resource-Poor Setting
Source: PLoS One. 2012 Dec 27;7(12):e52452. doi: 10.1371/journal.pone.0052452 (PMC3531465; doi:10.1371/journal.pone.0052452)
Supplement: Table S5 — (DOC) [file pone.0052452.s005.doc]

Table S5: Comparison of HIV-infected patients with CD4 count above 200 cells/ml vs. below 200 cells/ml in patients with Acinetobacter baumannii colonisation/infection admitted to adult ICUs

| Characteristic | HIV-infected patients with CD4>200cells/ml (N=8) | HIV-infected patients with CD4<200cells/ml (N=13) | P value |
| --- | --- | --- | --- |
| Age in years, median (IQR) | 28 (24-36) | 24 (21-27) | 0.013 |
| Male | 3 (37.5) | 4 (30.8) | 0.395 |
| For HIV infected: CD4 count, median (range) | 395 (267-570) | 78 (14-130) | <0.001 |
| Prior ICU admission, n (%) | 1 (12.5) | 2 (15.4) | 0.402 |
| Ward before coming to ICU, n (%) Medical A&E Trauma Unit Surgical wards Medical wards Gynaecology-Obstetrics wards Secondary hospital ICU Private hospital ICU TBH ICU | 3 (37.5) 0 (0) 3 (37.5) 0 (0) 0 (0) 2 (25) 0 (0) 0 (0) | 2 (15.4) 0 (0) 2 (15.4) 3 (23.0) 1 (7.7) 5 (38.5) 0 (0) 0 (0) | 0.041 |
| Intubated before coming to ICU, n (%) | 5 (62.5) | 11 (84.6) | 0.064 |
| Length of hospital stay in this admission before going to the ICU, median (IQR) | 7 (3-11) | 8 (4-13) | 0.602 |
| Recent surgery in this admission, n (%) | 2 (25) | 0 (0) | 0.083 |
| Admitted to hospital in the last six months before this ICU admission, n (%) | 3 (37.5) | 1 (7.7) | 0.032 |
| Timing of A.baumannii infection, median days after hospital admission (IQR) | 9 (6-12) | 8 (5-11) | 0.517 |
| Length of ICU stay in this admission in days, median (IQR) | 9 (5-19) | 11 (6-23) | 0.483 |
| Major adverse events in ICU, n (%) | 4 (50) | 11 (84.6) | <0.001 |
| Multi-organ dysfunction syndrome, n(%) | 5 (62.5) | 11 (84.6) | 0.002 |
| APACHE II score, n ± SD | 24.65 ± 8.93 | 42.31 ± 16.47 | <0.001 |
| Positive blood culture result for A.baumannii, n (%) | 4 (50) | 7 (53.8) | 0.382 |
| Positive tracheal aspirate culture result for A. baumannii, n (%) | 5 (62.5) | 9 (69.2) | 0.420 |
| Positive urine culture result for A.baumannii, n (%) | 0 (0) | 1 (7.7) | 0.105 |
| Positive A.baumannii culture from another site, n (%) | 0 (0) | 1 (7.7) | 0.119 |
| Deaths in ICU in this admission, n (%) | 3 (37.5) | 9 (69.2) | <0.001 |
